# Genetic polymorphisms of *SCN9A* are associated with oxaliplatin-induced neuropathy

**DOI:** 10.1186/s12885-016-3031-5

**Published:** 2017-01-19

**Authors:** María Sereno, Gerardo Gutiérrez-Gutiérrez, Juan Moreno Rubio, María Apellániz-Ruiz, Lara Sánchez-Barroso, Enrique Casado, Sandra Falagan, Miriam López-Gómez, María Merino, César Gómez-Raposo, Nuria Rodriguez-Salas, Francisco Zambrana Tébar, Cristina Rodríguez-Antona

**Affiliations:** 1 Medical Oncology Department, Infanta Sofía University Hospital, SS de los Reyes, Madrid, Spain; 2 Neurology Department, Infanta Sofía University Hospital, SS de los Reyes, Madrid, Spain; 3 Hereditary Endocrine Cancer Group, Spanish National Cancer Research Centre (CNIO), Madrid, Spain; 4 Medical Oncologist, Medical Oncology Department, La Paz University Hospital, Madrid, Spain; 5 Hereditary Endocrine Cancer Group, Human Cancer Genetics Programme, Spanish National Cancer Center (CNIO), Madrid, Spain; 6 ISCIII Center for Biomedical Research on Rare Diseases (CIBERER), Madrid, Spain

**Keywords:** Oxaliplatin neuropathy, Calcium channel, *SCN9A*

## Abstract

**Background:**

Oxaliplatin is a chemotherapy agent active against digestive tumors. Peripheral neuropathy is one of the most important dose-limiting toxicity of this drug. It occurs in around 60–80% of the patients, and 15% of them develop severe neuropathy. The pathophysiology of oxaliplatin neurotoxicity remains unclear. SCN9A is a gene codifying for a subtype sodium channel (type IX, subunit α) and mutations in this gene are involved in neuropathic perception. In this study we investigated whether *SCN9A* genetic variants were associated with risk of neurotoxicity in patients diagnosed of cancer on treatment with oxaliplatin.

**Methods:**

Blood samples from 94 patients diagnosed of digestive cancer that had received oxaliplatin in adjuvant or metastatic setting were obtained from three hospitals in Madrid. These patients were classified into two groups: “cases” developed oxaliplatin-induced grade 3–4 neuropathy (*n* = 48), and “controls” (*n* = 46) had no neuropathy or grade 1. The neuropathy was evaluated by an expert neurologist and included a clinical examination and classification according to validated neurological scales: *National Cancer Institute Common Toxicity Criteria* (*NCI-CTC*), *Oxaliplatin-Specific Neurotoxicity Scale (OSNS*) and *Total Neuropathy score (TNS*). Genotyping was performed for 3 *SCN9A* missense polymorphisms: rs6746030 (R1150W), rs74401238 (R1110Q) and rs41268673 (P610T), and associations between genotypes and neuropathy were evaluated.

**Results:**

We found that *SCN9A* rs6746030 was associated with protection for severe neuropathy (OR = 0.39, 95% CI = 0.16–0.96; *p* = 0.041). Multivariate analysis adjusting for diabetes provided similar results (*p* = 0.036). No significant differences in neuropathy risk were detected for rs74401238 and rs41268673.

**Conclusion:**

*SCN9A* rs6746030 was associated with protection for severe oxaliplatin-induced peripheral neuropathy. The validation of this exploratory study is ongoing in an independent series.

## Background

Oxaliplatin is a subtype of platinum drugs with significant activity against advanced or metastatic digestive tumors, mainly colorectal cancer (CRC) [1]. Peripheral neuropathy (PN) is a dose-limiting toxicity of oxaliplatin and classically induces two recognized forms of PN: acute and chronic [2].

Whereas acute PN is not dose-dependent, cumulative doses of oxaliplatin are related to occurrence of chronic peripheral neuropathy [3]. The acute neurotoxicity occurs in a high rate of oxaliplatin-treated patients, and it usually occurs during or within hours of infusion, and it is transient. The majority of the symptoms (including paresthesias and/or dysesthesias in the distal extremities and the perioral region) are typically induced by cold exposition and clinical features include distal sensory neuropathy. Chronic neuropathy is a dose-limiting toxicity defined as a cumulative sensory toxicity. The median dose for severe neuropathy is 780–850 mg/m^2^, and it is seen in 10–15% of patients [1]. It is a pure sensory, mostly axonal neuropathy with a stocking-and-glove distribution. Symptoms typically are presented between cycles and increase in intensity with cumulative dose. Several factors have been described to increase the risk of severe PN: previous neuropathies, Guillain-Barré syndrome, *diabetes mellitus*, anemia, hypoalbuminemia, hypomagnesemia and alcohol consumption, as well as have had a recent surgery [4]. However, a large part of the severe neuropathy cases remain unexplained and more studies are needed to discover the underlying factors and to identify patients with a high risk to develop severe PN.

Several markers have been described as potential predictors for acute and chronic oxaliplatin-induced neuropathy (OXLIN) [5–7]. However, none of these have been validated in prospective studies.

The voltage-gated sodium channels are presented in dorsal root ganglion (DRG) and sympathetic ganglion cells, as well as in their small-diameter peripheral axons. They are involved in the initiation and propagation of potentials. They act as gatekeepers of pain at peripheral nociceptors. Nine sodium channel subunits are described (Nav1.1-Nav1.9) [8]. *SCN9A* gene encodes Nav1.7 isoform. Mutations in *SCN9A* have been associated with different “channelopathies” with electrical hyperactivity of sensory neurons in dorsal root and a low reactivity of sympathetic ganglia neurons [9]. There are three human pain disorders related to these type of genetic disorders: a bi-allelic loss of function mutations (channelopathy-associated Insensitivity to Pain, CIP), and the opposite situation, an activating mutations with severe episodic pain in Paroxysmal Extreme Pain Disorder (PEPD) and Primary Erythromelalgia (PE) [10]. Moreover; a polymorphism in *SCN9A* gene (rs6746030) was associated with pain intensity perception in patients with different painful diseases, making DRG neurons more excitable as well as susceptible to aberrant pain perception [11]. Taking into account these evidences, we designed a study to explore the role of *SCN9A* common genetic variants in OXLIN development in patients diagnosed of digestive tumors that had undergone treatment with oxaliplatin.

## Methods

### Patient Selection and OXLIN evaluation

From 2012 to 2014, one hundred adults with a histologically confirmed diagnosis of digestive tumor (colon, rectum, gastric, pancreatic and biliary duct cancers in different stages) were recruited in three Spanish Hospitals (Infanta Sofía University Hospital, La Paz University Hospital and Infanta Leonor University Hospital). All patients had received an oxaliplatin based regimen (at least 780–800 mg/m^2^ of oxaliplatin dose) during the last 6 months previous to inclusion. Additional details about the type of tumor and treatment regimens are summarized in Table 1. All cancer patients were over 18 years; life expectancy of ≥3 months; Eastern Cooperative Oncology Group (ECOG) performance status of ≤2; adequate bone marrow, renal and hepatic function; and no previous history of neuropathy. The study was approved by La Paz University Hospital Ethic committee in December 2011 and all patients signed and gave written informed consent.

**Table 1:** Demographic and clinical characteristics of the series

Variables	Cases (TNS ≥ 15; *n* = 46) *N* (%)	Controls (TNS ≤ 6; *n* = 48) *N* (%)	*P* value*
Sex			ns
Female	14 (30%)	16 (33%)	
Male	32 (70%)	32 (67%)	
Age			ns
Median (min-max)	65 (41–83)	66 (36–82)	
Diabetes			**0.0055**
Yes	11 (24%)	2 (4%)	
No	35 (76%)	46 (96%)	
Hypothyroidism			ns
Yes	0 (0%)	0 (0%)	
No	46 (100%)	48 (100%)	
Tumor location			ns
Colon	23 (50%)	30 (63%)	
Rectum	9 (20%)	7 (15%)	
Biliary duct	2 (4%)	2 (4%)	
Pancreas	3 (6%)	4 (8%)	
Stomach	9 (20%)	5 (10%)	
Type of chemotherapy			ns
XELOX	35 (76%)	29 (61%)	
-Adjuvant	25	20	
-Palliative	10	9	
FOLFOX	6 (13%)	11 (23%)	
-Adjuvant	3	8	
-Palliative	3	3	
TOMOX	0 (0%)	2 (4%)	
-Adjuvant	0	0	
-Palliative	0	2	
FOLFIRINOX	1 (2%)	2 (4%)	
-Palliative	1	2	
EOX	4 (9%)	4 (8%)	
-Perioperative	4	4	
-Palliative	0	0	
Stage AJCC			ns
I-II	18 (39%)	16 (33%)	
III-IV	28 (61%)	32 (67%)	
Cumulative dose of oxaliplatin (mg)			ns
Median (min-max)	1616 (880–2045)	1624 (900–2050)	

*P values >0.05 are indicated as non significant (ns)

TNS: Total Neuropathy Score > 15

TNS: Total Neuropathy Score < 6

Inclusion criteria for cases were to have a digestive tumor; receiving oxaliplatin based regimen; cumulative doses 780–850 mg/m2, and the development of a severe neuropathy (NCI-CTC grade 3–4). Inclusion criteria for controls were also to have a digestive tumor; receiving oxaliplatin based regimen; cumulative dosis 780–850 mg/m2 and to have no neuropathy or mild neuropathy (NCI-CTC grade 0–1). Exclusion criteria for cases and controls was suffering a previous peripheral neuropathy.

The selected 100 patients were divided into two extreme groups: patients with a severe neuropathy and patients without neuropathy after receiving similar regimens and doses of oxaliplatin. This strategy was established to optimize the search of genetic differences between these opposite groups. According to this, fifty patients were defined as “cases”, those patients who developed an extreme and severe sensory neuropathy (defined as NCI-CTC grade 3); and fifty patients were defined as “controls”, those patients with no neuropathy or grade 1 neuropathy according to clinical scales (NCI-CTC grade 0–1) after receiving similar doses of oxaliplatin (Table 1).

To perform the clinical evaluation and the neuropathy grading in a homogeneous manner across the different collaborative centers, all patients included in the study underwent a comprehensive neurological examination by the same neurologist as well as neurophysiological studies, performing sural and peroneal conduction studies according to standardized protocols with a Nihon-Kohden electromyography and comparing them to normalized data from the EMG laboratory. The final classification of patients in cases and controls was performed according to the Total Neuropathy Score (TNS) and subscales scores (clinical and reduced, TNSc and TNSr). Cases were considered all those patients with a TNS > or = 15 and controls those patients with TNS value < or = 6 (Table 1). From the 100 patients recruited, 94 gave blood samples and completed a neurological evaluation and were included in the analysis.

### DNA extraction and SNP genotyping

FlexiGene DNA Kit (Qiagen) was used to isolate DNA from the blood samples of the patients. Final DNA analysis was made in 94 patients, 46 cases and 48 controls. Three SNPs in *SCN9A* (rs41268673, rs6746030 and rs74401238) were selected for genotyping using the KASPar SNP Genotyping System (LGC Genomics, UK) with 15 ng of genomic DNA. All assays included DNA samples with known genotypes and negative controls. The Sequence Detection System ABI PRISM® 7900HT (Applied Biosystems) was employed for fluorescence detection and allele assignment.

### Statistical analysis

Clinical variables among cases and controls were compared using Student *t*-test/Mann-Whitney *U* test (age and cumulative dose of oxaliplatin) or Chi^2^ test (gender, presence of *diabetes mellitus*, tumor type, chemotherapy regimen and tumor stage). Association between OXLIN (cases vs controls) and *SCN9A* polymorphisms was assessed using binary logistic regression analysis. In the multivariate binary logistic regression analysis, *diabetes mellitus* was included as covariate. We considered an additive genetic model for the SNPs evaluated, and when *P* value was <0.1, other genetic models were explored. Mann-Whitney *U* test was used to compare TNS grade between the different *SCN9A* genotypes. The SPSS software package v.19 was used for all statistical analyses. *P* values less than 0.05 were considered statistically significant.

## Results

The overall demographic and clinical characteristics of the series are represented in Table 1. All patients included received oxaliplatin treatment during at least 6 months (doses 780–850 mg/m2), according to the following protocols: eight courses of XELOX regimen: (Capecitabine) xeloda and oxaliplatin; 12 courses of FOLFOX regimen: 5-fluorouracil, folinic acid (leucovorin) and oxaliplatin; eight courses of TOMOX: raltitrexed and oxaliplatin; six courses of EOX: epirubicin, oxaliplatin and xeloda; or 12 courses of FOLFIRINOX: 5-fluorouacil, folinic acid, irinotecan and oxaliplatin.

The severity of the acute OXLIN was evaluated before every cycle and chronic or persistent toxicity was evaluated up to 3 months after last chemotherapy cycle. According to this, patients were classified in cases and controls according to TNS scale. We established that all those patients with a moderate or severe OXLIN (TNS score ≥ 15) were “cases” (46 patients) and those with inexistent or mild OXLIN (TNS ≤6) were “controls” (48 patients). The clinical characteristics of these patients were similar, except for *diabetes mellitus*, which was over-represented in the cases (Table 1).

Among the 94 patients studied, 41 (44%) patients presented “coasting effect” (that is, a worsening of neuropathy after finishing chemotherapy), 18 (20%) had dose reductions (around 25–30%) and 10 (12%) patients suspended chemotherapy because of the neuropathy. We found that all patients with severe acute OXLIN (TNS ≥15) also developed a chronic/cumulative disease.

### Association between OXLIN and *SCN9A* genotypes

With regards to genotyping, 3 *SCN9A* missense polymorphisms with a minimum allele frequency >2% in the European population (rs6746030 (p.R1150W), rs74401238 (p.R1110Q) and rs41268673 (p.P610T), were genotyped in the 94 patients. We identified 64 C/C, 29 C/T and 1 T/T patients for rs6746030 (p.R1150W). For rs41268673 (p.P610T) three individuals were heterozygous and the rest were homozygous wild type, and for rs74401238 (p.R1110Q) five were heterozygous and 89 were homozygous wild type.

We found that rs6746030 variant carriers had significantly lower neuropathy (OR = 0.39; 95% CI = 0.16–0.96; *p* = 0.041; dominant model) than wild-type patients. These findings suggest that *SCN9A* rs6746030 (p.R1150W) variant allele protects against the development of moderate-severe OXLIN (Table 2). Among the different clinical factors analyzed (gender, age, *diabetes mellitus*, type of tumor, chemotherapy regimen) only *diabetes mellitus* was significantly associated with moderate-severe OXLIN (OR = 7.23; 95% CI = 1.51–34.73; *p* = 0.014) (Table 3). Multivariate analysis adjusting for *diabetes mellitus* did not substantially changed the association of *SCN9A* rs6746030 polymorphism with OXLIN (OR = 0.36; 95% CI = 0.14–0.94; *p* = 0.036) (Table 3).

**Table 2:** *SCN9A* SNPs included in the study with genotype frequencies among cases and controls

SNP	Total nr. of patients	Cases TNS ≤ 6 (*n* = 48)	Controls TNS ≥ 15 (*n* = 46)	*P* value*
rs6746030				**0.041**
C/C	64	23 (48%)	41 (89%)	
C/T	29	24 (50%)	5 (11%)	
T/T	1	1 (2%)	0 (0%)	
rs41268673				0.589
C/C	91	46 (96%)	45 (98%)	
C/A	3	2 (4%)	1 (2%)	
A/A	0	0 (0%)	0 (0%)	
rs74401238				0.683
G/G	89	45 (94%)	44 (98%)	
G/A	5	3 (6%)	2 (2%)	
A/A	0	0 (0%)	0 (0%)	

*Univariate binary logistic regression analysis

**Table 3:** Association of rs6746030 with OXLIN under a dominant genetic model

Variable	OR	95% CI	*P* value
rs6746030 univariate	0.39	0.16–0.96	0.041
rs6746030 multivariate^a^	0.36	0.14–0.94	0.036
*Diabetes mellitus*	7.23	1.51–34.73	0.014

^a^Multivariate binary logistic regression analysis adjusting for *diabetes mellitus*

When TNS grade was compared with rs6746030 genotype, in multivariable analysis adjusting by *diabetes mellitus,* the variant carriers had protection against OXLIN (OR = 0.89; 95% CI = 0.81–0.99; *P* = 0.044).

The other two *SCN9A* SNPs studied were not associated with OXLIN development in the population included in the study (Table 2). However, the low number of carriers for *SCN9A* rs74401238 (p.R1110Q) and rs41268673 (p.P610T) indicates a low statistical power in the analysis.

## Discussion

Oxaliplatin peripheral neuropathy affects a large number of patients and can lead to treatment suspension. Most patients recover from the neuropathy in a variable period of time, but long-term nerve damage can also occur, compromising the quality of life of these patients [12, 13]. Several studies have tried to explain OXLIN mechanism, but, so far, the molecular bases for it remain unknown. In this study we proposed that alterations in Na channel structure could increase susceptibility for OXLIN development. Previous studies have shown that oxaliplatin causes a functional channelopathy induced by oxalate affecting channels located in cellular membrane [14]. Several preclinical studies with animal models suggested that oxaliplatin modulates the axonal voltage-gated sodium channels inducing a slow sodium channel kinetics, shifting the voltage dependence to more negative potentials [15]. However, it is not clear what are the mechanisms that justify the development of an acute “sodium channelopathy” in oxaliplatin-treated patients, with inconsistent in vitro studies concerning the role of transient and persistent Na + conductances [16]. In addition to the “channelopathy hypothesis”, other studies have described the “oxidative hypothesis”, supported by primary cultures of astrocytes activated in vivo by oxaliplatin treatment. According to the hypothesis of channel disorders induced by oxaliplatin, OXLIN happens when oxaliplatin is accumulated in dorsal root ganglia (DRG) cells. This event, as well as mitochondrial dysfunction with alteration in redox metabolism, is able to induce DRG cells apoptosis [17, 18].

Diabetes has been related to a higher risk to develop neuropathy with different anticancer drugs. Kus T et al. demonstrated in a retrospective analysis that paclitaxel-based therapy in diabetic patients with breast cancer, diabetes duration above 5 years could affect the incidence and severity of PSN without known baseline neuropathy [19]. De la Morena et al. found similar findings in patients with breast cancer treated with paclitaxel every week. They demonstrated that preexisting diabetes was associated with long-lasting significant neuropathy [20]. As well as with Taxol, several authors have described that diabetic patients are more vulnerable to develop OXLIN. Uwahn AN et al. found that although the presence of diabetes did not appear to affect the severity of OXLIN, patients with diabetes could develop OXLIN with lower doses of oxaliplatin (*P* < .05) [21].

However, no molecular studies have identified markers able to predict OXLIN development. *Argyriou A* et al. found polymorphisms in different voltage-gated sodium channels with a potential role in predicting severe acute and chronic OXLIN. They found that the experimental model of the skeletal muscle sodium channel *SCN4A* rs2302237 and the tetrodotoxin-resistant *SCN10A* rs1263292 polymorphisms could be related to the development of acute OXLIN [22, 23].

*SCN9A* encodes Nav1.7 isoform and it plays an important role in defining of threshold for excitation of nociceptors and can control neurotransmitter release at the terminals of these receptors. Homozygous loss of function mutations in Na_v_1.7 has been related to a congenital inability to suffer pain and anosmia in men. In addition, heterozygous gain of function mutations have been described in different clinical pain syndromes of inherited erythromelalgia (IEM: pain and erythema exacerbated by warming), paroxysmal extreme pain disorder (PEPD: proximal pain and autonomic features in ocular/mandibular and sacral regions), and small fiber neuropathy (SFN: degeneration of small diameter sensory and autonomic axons presenting with a severe burning pain) [24]. As we already have mentioned, different mutations in *SCN9A* gene could cause chronic pain and pain insensitivity syndromes [25]. For example, *Peddareddygari LR* et al. demonstrated that homozygous p.G2755T mutation in exon 15 of this gene is associated with congenital insensitivity to pain (CIP) [26, 27]. Other authors have related some mutations like p.L1612P with a Cold Sensitive Paroxysmal Extreme Pain Disorder [28]. According to these data and the role of this gene in pain and cold perception, we decided to analyzed *SCN9A* missense SNPs (rs6746030 (p.R1150W), rs74401238 (p.R1110Q) and rs41268673 (p.P610T)) as a potential cause of genetic susceptibility for a cold-induced neuropathy like OXLIN. We found that *SCN9A* rs6746030 variant allele protected against the development of moderate-severe OXLIN in univariate and multivariate analysis (*p* = 0.041 and *p* = 0.036, respectively).

A validation in a prospective series of patients receiving oxaliplatin is needed to confirm this association. We could not obtain any conclusions about the other two SNPs because of low frequency in European population.

In addition, we analyzed the correlation between chronic and acute OXLIN. We observed that all patients considered “cases”, with severe acute OXLIN, also had a moderate-severe chronic/cumulative neuropathy. *Argyriou* et al. also demonstrated this correlation in a cohort of patients with OXLIN. A possible explanation for this could be the cellular stress affecting the sensory cells as a result of the prolonged activation of SCNAs, inducing a decrease of neuron metabolism and axoplasmic function in the DRG cells [18]. Preclinical data with sural nerve cultures exposed to oxaliplatin showed alterations in sodium channel kinetics and prolonged opening of sodium channels resulting in an increase in sodium currents [29].

## Conclusion

In conclusion, OXLIN is a multifactorial disease. *Diabetes mellitus* and previous neuropathy increase the risk for developing OXLIN; however, so far, no genetic predisposition markers have been defined. In this study, we found that *SCN9A* rs6746030 (p.R1150W) variant allele is associated with a protective effect against developing moderate-severe OXLIN. Prospective evaluation of these findings is needed to confirm the role of this *SCN9A* polymorphism in OXLIN development.

## Abbreviations

CIP: Channelopathy associated insensitivity to pain
CIP: Congenital Insentitivity to pain
CRC: Colorectal cancer
DRG: Dorsal root ganglia
ECOG: Eastern Cooperative Oncology Group
IEM: Inherited erythromelalgia
NCI-CTC: National Cancer Institute-Common Toxicity Criteria
OSNS: Oxaliplatin Specific Neuropathy Scale
OXLIN: Oxaliplatin Induced neuropathy
PD: Paroxysmal Extreme Pain Disorders
PE: Primary Erithromelalgia
PEDP: Paroxysmal extreme pain disorder
PN: Peripheral neuropathy
SFN: Small fiber neuropathy
TNS: Total Neuropathy Scale
